# FACTORS INFLUENCING SELF-REPORTED COGNITION OVER TIME

**DOI:** 10.1093/geroni/igz038.804

**Published:** 2019-11-08

**Authors:** Nikki Hill, Mindy Katz

**Affiliations:** 1 Penn State University, University Park, Pennsylvania, United States; 2 Albert Einstein College of Medicine, Bronx, New York, United States

## Abstract

Self-reported cognitive problems among cognitively intact older adults are often associated with an increased risk of future cognitive decline and Alzheimer’s disease (AD). However, cross-sectional evidence suggests that self-reported cognition may be more influenced by factors such as personality or affective symptoms than concurrent objective cognitive performance. Furthermore, self-reported cognition is measured using a variety of items that assess different constructs (e.g., current memory performance, perceived decline over time), which may be differentially influenced by individual characteristics or item interpretation. The purpose of this symposium is to present findings from multiple analyses that examined the influence of individual characteristics (i.e., personality, perceived stress, and family history of dementia) on self-reported cognitive problems, and to further describe how item type influences older adults’ responses to questions about their memory. First, we present the results of an investigation that examined the influence of personality on three types self-reported memory, with a specific focus on how these associations may differ in Black and White older adults. Second, we extend this discussion with results of an examination of associations among personality, family history of AD, and memory self-report. Our third presentation explores bidirectional associations between perceived stress and memory complaints over time. And finally, we present the results of a factor analysis of self-reported cognition items that distinguishes those that tend to travel together over time from those that are better at discriminating between individuals.

